# Tophus burden reduction with pegloticase: results from phase 3 randomized trials and open-label extension in patients with chronic gout refractory to conventional therapy

**DOI:** 10.1186/ar4318

**Published:** 2013-09-26

**Authors:** Herbert SB Baraf, Michael A Becker, Sergio R Gutierrez-Urena, Edward L Treadwell, Janitzia Vazquez-Mellado, Claudia D Rehrig, Faith D Ottery, John S Sundy, Robert A Yood

**Affiliations:** 1 Center for Rheumatology & Bone Research, 2730 University Blvd West, Wheaton, MD 20902, USA; 2 The University of Chicago, 5801 South Ellis Ave, Chicago, IL 60637, USA; 3 Hospital Civil de Guadalajara, Independencia Oriente, Guadalajara, Jalisco, Mexico; 4 East Carolina University, 1001 East Fifth Street, Greenville, NC 27858, USA; 5 Hospital General de Mexico, Dr. Balmis 148, Doctores, Cuauhtemoc, 06726 Mexico City, Federal District, Mexico; 6 Savient Pharmaceuticals, Inc, 400 Crossing Blvd, Bridgewater, NJ 08807, USA; 7 Formerly of Savient Pharmaceuticals, Inc, 400 Crossing Blvd, Bridgewater, NJ 08807, USA; 8 Duke University Medical Center, 2301 Erwin Road, Durham, NC 27705, USA; 9 Reliant Medical Group, 630 Plantation Street, Worcester, MA 01605, USA

## Abstract

**Introduction:**

Two replicate randomized, placebo-controlled six-month trials (RCTs) and an open-label treatment extension (OLE) comprised the pegloticase development program in patients with gout refractory to conventional therapy. In the RCTs, approximately 40% of patients treated with the approved dose saw complete response (CR) of at least one tophus. Here we describe the temporal course of tophus resolution, total tophus burden in patients with multiple tophi, tophus size at baseline, and the relationship between tophus response and urate-lowering efficacy.

**Methods:**

Baseline subcutaneous tophi were analyzed quantitatively using computer-assisted digital images in patients receiving pegloticase (8 mg biweekly or monthly) or placebo in the RCTs, and pegloticase in the OLE. Tophus response, a secondary endpoint in the trials, was evaluated two ways. Overall tophus CR was the proportion of patients achieving a best response of CR (without any new/enlarging tophi) and target tophus complete response (TT-CR) was the proportion of all tophi with CR.

**Results:**

Among 212 patients randomized in the RCTs, 155 (73%) had ≥1 tophus and 547 visible tophi were recorded at baseline. Overall tophus CR was recorded in 45% of patients in the biweekly group (*P* = 0.002 versus placebo), 26% in the monthly group, and 8% in the placebo group after six months of RCT therapy. TT-CR rates at six months were 28%, 19%, and 2% of tophi, respectively. Patients meeting the primary endpoint of sustained urate-lowering response to therapy (responders) were more likely than nonresponders to have an overall tophus CR at six months (54% vs 20%, respectively and 8% with placebo).

Both overall tophus CR and TT-CRs increased with treatment duration in the OLE, reaching 70% (39/56) of patients and 55% (132/238) of target tophi after one year of treatment in patients receiving pegloticase during both the RCTs and OLE. At that time point, more tophi had resolved in responders (102/145 or 70% of tophi) than nonresponders (30/93; 32%).

**Conclusions:**

Pegloticase reduced tophus burden in patients with refractory tophaceous gout, especially those achieving sustained urate-lowering. Complete resolution of tophi occurred in some patients by 13 weeks and in others with longer-term therapy.

**Trial registrations:**

NCT00325195, NCT01356498

## Introduction

*Refractory gout* refers to the condition of a population of patients with symptomatic gout in whom treatment has failed to maintain a serum uric acid (SUA) level less than 6 mg/dl with oral urate-lowering therapies (ULTs) and appropriate medical management [1,2]. Patients with refractory gout are at risk for progressive urate crystal deposition disease, characterized by frequent attacks of acute gouty arthritis, gouty arthropathy and enlarging tophi, which are often associated with chronic pain, impairment of physical function and compromised health-related quality of life [1,3,4].

The tophus, a cardinal feature of chronic gout, is a mass of urate crystals embedded in fibrous and inflammatory tissue. Tophi contribute to gouty joint destruction and deformity and may undergo acute or chronic ulceration, erode adjacent bone, cause pressure effects on surrounding tissues and organs, interfere with joint function or become infected [1,5-9]. Once established, tophi do not regress or resolve unless the extracellular urate saturation that supports urate crystal deposition (reflected by hyperuricemia or SUA in excess of 6.8 mg/dl) is reversed and subsaturating urate levels are maintained. Achievement and maintenance of SUA in a range less than 6.0 mg for months to years does, however, promote dissolution of urate crystal deposits and prevent further crystal deposition in tissues [10]. Rates of resolution of tophaceous deposits appear to be dependent on the extent of urate-lowering [11,12]. Furthermore, tracking the course of tophus size or number over time during treatment provides a means by which to assess the clinical benefit of urate-lowering agents and even their disease-modifying capability.

With currently prescribed doses of available oral ULTs, tophus resolution commonly requires many months to years [13,14]. Patients with tophaceous gout and chronic pain, functional impairment, compromised quality of life or complications of gouty deformity have a compelling need for rapid reversal of their tophaceous disease.

Pegloticase is a mammalian PEGylated recombinant uricase approved in the United States for the treatment of chronic gout refractory to conventional therapy and in the European Union for severe debilitating chronic tophaceous gout in patients who may also have erosive joint involvement. The efficacy of pegloticase in reducing and maintaining plasma uric acid (PUA) levels substantially below 6 mg/dl was initially demonstrated in a randomized, open-label phase II study [2]. Two patients in the phase II trial were documented with marked reductions in tophus size after treatment with pegloticase for 12 weeks, which coincided with sustained decreases in PUA to less than 2 mg/dl [15]. On the basis of this unprecedented finding, tophus reduction or resolution was evaluated as a key secondary endpoint in two replicate, randomized, six-month, double-blind, placebo-controlled phase III trials of pegloticase in patients with chronic gout refractory to conventional therapy. The primary results of these randomized controlled trials (RCTs) have been reported previously [16]. This paper provides a detailed analysis of the tophus response to pegloticase in the RCT population and in a subsequent open-label extension (OLE) study enrolling the majority (96%) of RCT completers.

## Methods

### Patients

Eligible patients were ages 18 years or older, had baseline SUA levels of 8 mg/dl or higher and at least one of the following clinical features: three or more self-reported gout flares during the previous eighteen months, one or more tophi or gouty arthropathy. They also had contraindications for or intolerance to treatment with allopurinol (82%; 174 of 212 patients) or a failure to achieve a normal SUA after at least three months of treatment with the maximum medically appropriate dose of allopurinol (the only xanthine oxidase inhibitor approved during the trials). Key exclusion criteria included glucose-6-phosphate dehydrogenase deficiency, pregnancy, unstable angina, uncontrolled cardiac arrhythmia, uncompensated congestive heart failure, uncontrolled hypertension (blood pressure higher than 150/95 mmHg on medication), ongoing maintenance dialysis or a history of solid organ transplant.

### Trial design and randomization

The design of the replicate RCTs has been reported previously [16]. Briefly, eligible patients were randomized in a 2:2:1 ratio to receive intravenous infusions of 8 mg pegloticase every two weeks (biweekly treatment), 8 mg of pegloticase alternating with placebo (monthly treatment) or placebo at each infusion for twenty-four weeks. Randomization was stratified based upon the presence or absence of tophi. Patients receiving ULT at the time of screening underwent a one-week washout period. All patients received prophylaxis for both gout flares and infusion reactions [16].

The primary efficacy endpoint in the RCTs was the proportion of PUA responders in the pegloticase-treated versus placebo-treated groups, with *response* defined as patients who maintained PUA level less than 6 mg/dl for 80% or more of the time during months 3 and 6. Patients who discontinued treatment prematurely for any reason were deemed PUA nonresponders.

Patients who completed treatment in either of the RCTs were eligible to participate in an OLE study. At the patient’s final visit in the RCT, while still blinded to treatment assignment and UA response, the investigator and patient decided whether pegloticase would be administered biweekly or monthly during the OLE study. A change in dosing frequency (monthly to biweekly, or biweekly to monthly) was allowed once after week 25 of the OLE and a second time after the RCT results were unblinded. The OLE study was initially designed as a 12-month study. Protocol amendments allowed treatment with pegloticase for a maximum of 31 months. The trial protocols were approved by a central institutional review board (IntegReview, Austin, TX, USA) and local institutional review boards, and informed consent and Health Insurance Portability and Accountability Act of 1996 documentation were obtained prior to any study-related procedures.

### Tophus assessment

Tophus assessment was conducted using Computer-Assisted Photographic Evaluation in Rheumatology (CAPER) methodology [17]. Two assessments (overall tophus complete response (CR) and target tophus CR) were prespecified as secondary efficacy endpoints in the RCTs and as secondary clinical outcomes in the OLE study. Each study site was supplied with a calibrated digital camera, digital media storage cards, preprinted templates for hand and foot photography and a light stand. A designated individual was trained and then assumed responsibility for taking all photographs at each site. Photographs of the hands and feet were obtained for all patients at baseline. Photographic evaluation was repeated at weeks 13, 19 and 25 of the RCTs and at weeks 13, 25, 53, 77 and 101 of the OLE study (for patients with tophi who remained in the trials) using the same views of the tophus sites identified at baseline. Additional serial photographs of up to two other anatomic regions were taken at the discretion of the investigator based on additional tophi identified at the baseline visit.

Photographs on digital media cards were sent to RadPharm (Princeton, NJ, USA), where central readers (board-certified rheumatologist and internist), who were blinded to treatment assignment, evaluated the photographs prospectively and identified sites of tophi present at the start of treatment. Central readers chose up to seven target tophi (five measurable tophi and two unmeasured tophi) for each patient for assessment throughout the study. Tophi were considered “measurable” if they had distinguishable borders 5 mm or larger in the longest dimension at baseline. Tophi were considered “unmeasurable” if they could not be measured accurately because of their location, shape or other factors, but could be assessed qualitatively. To be included for assessment, unmeasured tophi were approximated to be 10 mm or greater at the longest dimension.

The size of each target tophus was measured using digital photographs and MedStudio® image analysis software (Megasoft, Ltd, Hyderabad, India). Reading was performed using a sequential locked-read format whereby the reader could neither make changes to past time points nor view subsequent time points. For each measurable target tophus, size was determined in two dimensions with electronic calipers using the diameter and the longest width perpendicular to the diameter. Response to treatment was categorized on the basis of change from baseline as complete response (CR; 100% decrease in area of the tophus), marked response (MR; 75% or greater to less than 100% decrease), partial response (PR; 50% or greater to less than 75% decrease), stable disease (SD; less than 50% decrease to less than 25% increase) and progressive disease (PD; 25% or greater increase). For each unmeasured target tophus, response to treatment was semiquantitatively assessed on the basis of the impression of the central reader, and response was categorized as CR, improved, SD or PD.

**Figure 1:**
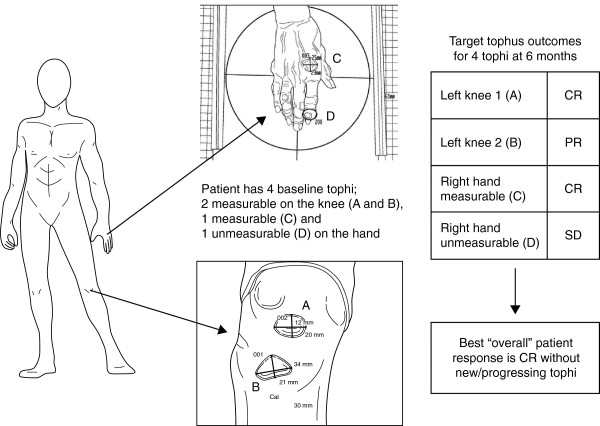
**Illustration of a hypothetical patient showing the target tophus responses and overall tophus response.** The diagram shows the tophus scoring system for a sample patient with four baseline tophi (three measurable and one unmeasurable). Tophus area was defined for each measurable tophus using the longest dimension and its perpendicular axis. The circle marks the unmeasurable tophus. Each baseline target tophus was rescored at all subsequent study visits. The patient illustrated in the figure had a best “overall” response of complete response (CR) because the patient had at least one target tophus response of CR and no new or progressing tophi at any time during the study. PR, partial response; SD, stable disease.

Tophus resolution was a secondary endpoint in the RCTs. The principal assessment was the proportion of patients achieving a *tophus CR*, defined as complete resolution of at least one tophus without development of new tophi or progressive enlargement of any other tophus. This measurement of a patient’s single **overall** best tophus response was intended to control for patients with multiple tophi and varying responses (Figure 1). If any new or progressing tophi were recorded at any time during the study, the final overall tophus response for that patient was PD. A second measure of tophus resolution was based on the total number of photographically identified baseline tophi. *Target tophus CR* (TT-CR) was defined as 100% decrease in the area of the tophus and is reported as the number or proportion of all baseline tophi with CR at the specific study visit.

### Statistical analysis

The two tophus assessment endpoints are presented as pooled data for the modified intent-to-treat (mITT) population in the RCTs, which comprised all randomized patients who received at least one dose of study medication and had at least one postdose observation. Only patients with tophi were included in these analyses (tophus-evaluable population). The number or proportion of patients with overall best response of CR was compared for each pegloticase dosing group versus the placebo group using Fisher’s exact test. A similar analysis was applied to the number of tophi showing CR for the TT-CR endpoint. The overall tophus CR was further analyzed according to the patient’s PUA response, which was the predefined primary endpoint of the RCTs (lower than 6 mg/dl for 80% of the time during months 3 and 6).

## Results

### Baseline tophus status

**Figure 2:**
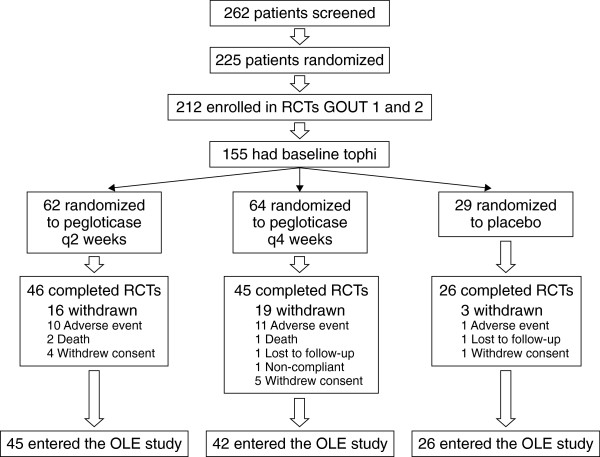
**Consolidated Standards of Reporting Trials Statement (CONSORT) diagram for the pooled tophus-evaluable population.** This population flowchart shows the disposition of all patients with baseline tophi pooled from the two randomized, placebo-controlled trials of pegloticase known as GOUT 1 and GOUT 2. OLE, open-label extension; RCT, randomized controlled trial.

The mITT population included 212 patients with a mean gout duration of 15 years. From among these patients, 155 (73%) (Figure 2) had at least one baseline tophus (Table 1). Baseline characteristics for tophi were well-balanced across the three treatment arms of the RCTs, and no differences were noted in baseline demographics for patients with and without tophi (Table 2). During the RCTs, 21 patients showed a new incident tophus (all 21 patients also had baseline tophi). New tophi were seen in 6% (4 of 62), 11% (7 of 64) and 35% (10 of 29) of patients receiving biweekly pegloticase, monthly pegloticase and placebo treatment, respectively.

**Table 1:** Frequency and characteristics of baseline tophi for patients in the randomized trials

**Characteristics**	**Pegloticase biweekly**	**Pegloticase monthly**	**Placebo**
	**(*n *** **= 85)**	**(*n *** **= 84)**	**(*n *** **= 43)**
Patients with one or more baseline tophi, *n* (%)	62 (73)	64 (76)	29 (67)
Total number of measurable baseline tophi	159	142	83
Median target area of measurable target tophi, mm^2^ (range)	378 (25 to 4,080)	377 (25 to 10,625)	460 (30 to 6,230)

**Table 2:** **Baseline characteristics for patients with and without subcutaneous tophi**^**a**^

**Demographics**	**Patients with**	**Patients without**
	**baseline tophi**	**baseline tophi**
	**(*n *** **= 155)**	**(*n *** **= 57)**
Males, *n* (%)	126 (82)	47 (81)
Whites, *n* (%)	106 (68)	37 (65)
Mean age, years (±SD)	56.7 (13.7)	51.9 (14.4)
Mean BMI, kg/m^2^ (±SD)	31.3 (6.6)	36.7 (8.6)
Mean baseline PUA, mg/dl (±SD)	9.8 (2.9)	9.4 (3.3)

^a^BMI, body mass index; PUA, plasma uric acid.

### Overall tophus complete response

At each time point, a significantly higher proportion of patients treated with biweekly pegloticase achieved CR compared with placebo (*P* ≤ 0.011) (Table 3). By the first postbaseline assessment in the RCTs (13 weeks), 22% of patients treated with biweekly pegloticase had attained an overall tophus CR, compared with 0% of patients in the placebo group (*P* = 0.011). At the final visit, 40% of patients had achieved a best response of CR compared with 7% in the placebo group (*P* = 0.002). Although a higher proportion of patients treated with monthly pegloticase also achieved an overall tophus CR compared to placebo-treated patients at each time point, these differences did not reach statistical significance (Table 3).

**Table 3:** **Overall tophus complete response in patients over time during the randomized controlled trials**^**a**^

**Variables**	**Pegloticase**	**Pegloticase**	**Placebo**
	**biweekly**	**monthly**	
	**(*n *** **= 62)**	**(*n *** **= 64)**	**(*n *** **= 29)**
Week 13			
Patients with evaluable tophi at visit, *n*	46	48	25
CR, *n* (%)	10 (22)	4 (8)	0
*P* value^b^	0.011	0.292	–
Week 25			
Patients with evaluable tophi at visit, *n*	40	39	24
CR, *n* (%)	18 (45)	10 (26)	2 (8)
*P* value^b^	0.002	0.110	–
Final visit^c^			
Patients with evaluable tophi, *n*	52	52	27
CR, *n* (%)	21 (40)	11 (21)	2 (7)
*P* value^b^	0.002	0.200	–

^a^CR, complete response. ^b^Pegloticase groups versus placebo for CR. ^c^Final visit includes data from the patient’s final visit, whenever it occurred, for all evaluable completers and noncompleters.

### Target tophus complete response

**Figure 3:**
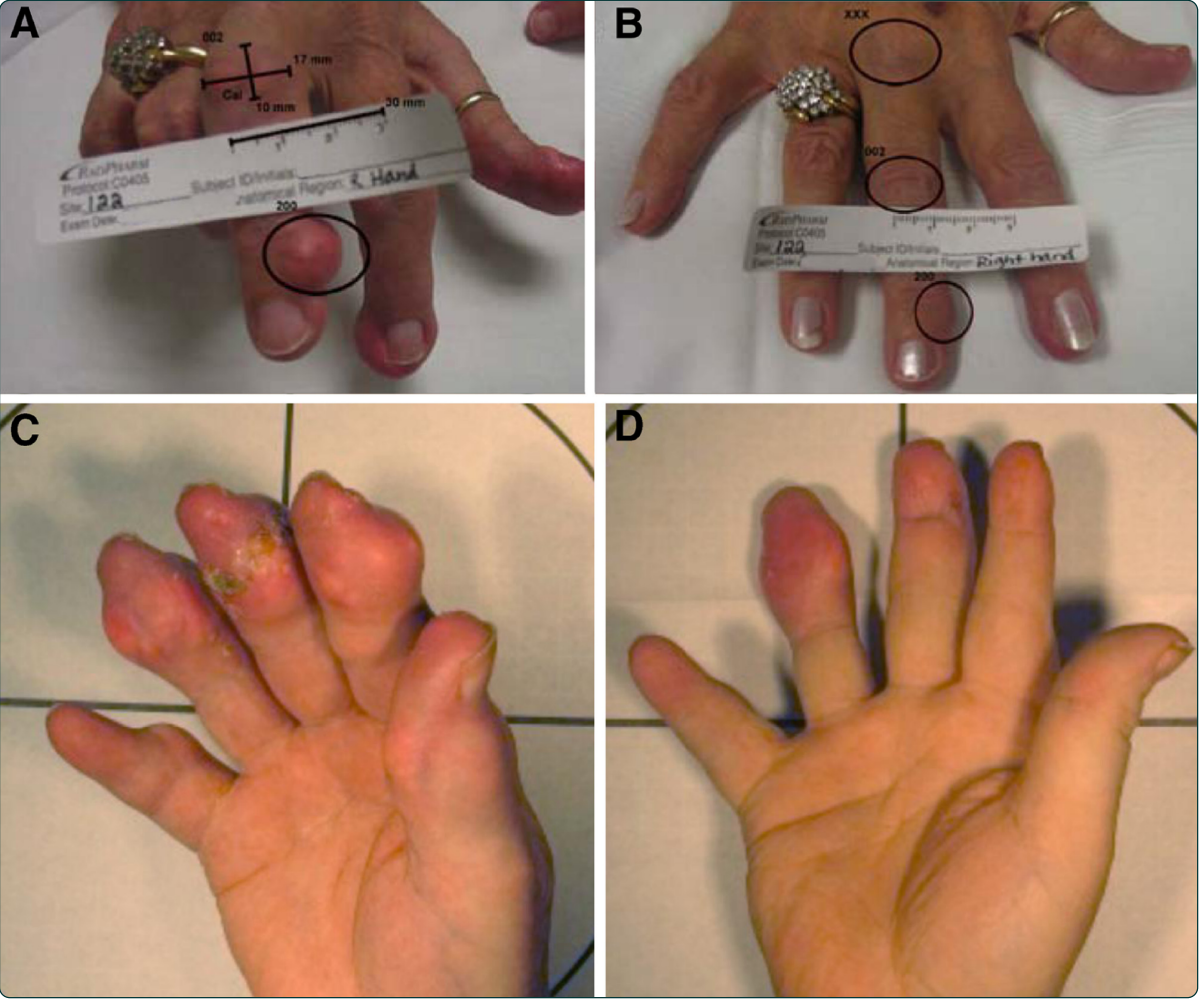
**Digital images showing complete resolution of tophi in two patients who received pegloticase biweekly. (A)** Photograph of patient 1 showing baseline tophus on the medial aspect of the patient’s right third distal interphalangeal (DIP) joint. **(B)** Photograph of patient 1 showing complete resolution of target tophus at week 13. Note also the reduction in the area of a large tophus on the third proximal interphalangeal joint. **(C)** Photograph of patient 2 showing baseline tophi on the patient’s right thumb; on the second, third and fourth DIP joints with ulcerations; and on the fifth proximal interphalangeal joint after six months of treatment with placebo in the randomized controlled trials. **(D)** Photograph of patient 2 showing complete resolution of target tophi on four digits after 25 weeks of treatment with twice-monthly pegloticase in the open-label extension study.

Fifty-seven (25%) of two hundred twenty-nine baseline target tophi in the biweekly pegloticase group achieved CR as assessed at the final (six-month) RCT study visit (Table 4). In comparison, TT-CR was achieved in 30 (15%) of 201 target tophi with monthly pegloticase and in 2 (2%) of 117 target tophi with placebo treatment. Photographs of CRs in patients treated with pegloticase biweekly are shown in Figure 3.

**Table 4:** **Individual target tophus response categories at patient’s final visit in the randomized trials**^**a**^

**Categories**	**Pegloticase**	**Pegloticase**	**Placebo**
	**biweekly**	**monthly**	
	**(*n *** **= 62)**	**(*n *** **= 64)**	**(*n *** **= 29)**
Total number of tophi^b^	229	201	117
Response category, *n* (%)			
CR	57 (25)	30 (15)	2 (2)
PR or MR	49 (21)	33 (16)	12 (10)
SD	111 (49)	121 (60)	89 (76)
PD	1 (0.4)	1 (0.5)	3 (3)
Unable to evaluate	11 (5)	16 (8)	11 (9)

^a^CR, complete response; MR, marked response; PD: progressive disease; PR, partial response; SD: stable disease. ^b^Total tophi includes both measurable and unmeasurable tophi.

### Tophus resolution by plasma uric acid responder status in the randomized trials

Tophus responses were analyzed on the basis of PUA responder status. Forty-two percent of patients in the biweekly pegloticase group and thirty-five percent of those in the monthly pegloticase group met the primary endpoint of the RCT and qualified as PUA responders [16]. Overall, tophus CR occurred more frequently at the six-month visit among PUA responders than among nonresponders: 62% (13 of 21 patients) versus 26% (5 of 19 patients) in the biweekly pegloticase group and 44% (7 of 16 patients) versus 15% (4 of 26 patients) in the monthly pegloticase group. When evaluated by response status and independent of dosing regimen, CR was documented in 54% of responders (20 of 37 patients) and in 20% of nonresponders (9 of 45 patients) after six months of RCT treatment.

### Tophus evaluation during open-label extension study

A total of 113 patients with tophi at the baseline assessment of the RCTs entered the OLE study, including 45 patients from the biweekly pegloticase group, 42 patients from the monthly pegloticase group and 26 patients from the placebo group. Patients in the OLE study were categorized according to their treatment regimen and response status in the RCTs (Table 5).

**Table 5:** **Overall tophus complete response and target tophus complete response for patients in the open-label extension study**^**a**^

**Visit during**	**Biweekly pegloticase in**	**Monthly pegloticase in**	**Placebo in RCT and**	**All patients**^**c**^
**OLE parameter**^**b**^	**RCT and continued**	**RCT and continued**	**initiated pegloticase**	
	**pegloticase in OLE**	**pegloticase in OLE**	**in OLE**	
	**(*n *** **= 45)**	**(*n *** **= 42)**	**(*n *** **= 26)**	**(*N *** **= 113)**
Overall CR, *n*/*N* (% patients)				
Week 13 visit	17 of 36 (47)	12 of 28 (43)	7 of 16 (44)	36 of 80 (45)
Week 25 visit	20 of 31 (65)	16 of 28 (57)	11 of 15 (73)	47 of 74 (64)
Week 53 visit	21 of 29 (72)	18 of 27 (67)	11 of 12 (92)	50 of 68 (74)
Week 77 visit	17 of 27 (63)	12 of 19 (63)	10 of 11 (91)	39 of 57 (68)
Week 101 visit	3 of 5 (60)	3 of 3 (100)	2 of 2 (100)	8 of 10 (80)
Final visit	23 of 39 (59)	20 of 34 (59)	13 of 21 (62)	56 of 94 (60)
TT-CR, *n*/*N* (% tophi)				All target tophi (*N* = 357)
Week 13 visit	55 of 164 (34)	36 of 104 (35)	17 of 89 (19)	108 of 357 (30)
Week 25 visit	67 of 136 (49)	52 of 103 (50)	39 of 79 (49)	158 of 318 (50)
Week 53 visit	70 of 138 (51)	62 of 100 (62)	53 of 64 (83)	185 of 302 (61)
Week 77 visit	85 of 138 (62)	40 of 61 (66)	51 of 62 (82)	176 of 261 (67)
Week 101 visit	4 of 9 (44)	10 of 10 (100)	8 of 12 (67)	22 of 31 (71)
Final visit	90 of 164 (55)	68 of 121 (56)	49 of 105 (47)	207 of 390 (53)

^a^CR, complete response; OLE, open-label extension study; RCT, randomized controlled trial; TT-CR, target tophus complete response. ^b^Values shown are number of patients (*n*) with overall tophus response-CR/total number of patients (*N*) evaluated at the visit or number of target tophi (*n*) with CR/total number (*N*) of target tophi evaluated at the visit. ^c^This population included all tophus-evaluable patients at each time point, both PUA responders and nonresponders, as defined by the primary endpoint in the RCTs.

The proportion of patients with an overall response of CR and the proportion of tophi with TT-CR increased with treatment duration in the OLE study, reaching (39 (70%) of 56 patients and 132 (55%) of (238) of target tophi after one year of treatment in patients receiving pegloticase during both studies. At the final OLE study visit, among patients randomized to the biweekly dose, overall tophus CR was achieved in 83% of RCT responders (19 of 23 patients) compared with 25% of RCT nonresponders (4 of 16 patients). Comparable results were found in the analysis of TT-CR (Table 5). For all patients in the OLE study, overall tophus CR was achieved in 60% of patients (56 of 94) and the TT-CR was 53% (207 of 390 target tophi) at the final visit. Among responders to pegloticase in the RCTs, 79% (123 of 156) of target tophi had resolved (compared with 27% in nonresponders; 35 of 129) by the patient’s final visit of the OLE study. Among patients initially randomized to placebo in the RCTs, 13 (62%) of 21 patients had an overall tophus CR with either biweekly or monthly pegloticase by their final visit in the OLE study.

### Tophus size and response in the open-label extension study

To assess the relationship between tophus size and response in individual tophi, the TT-CR was evaluated categorically by tophus area throughout the OLE period. Numerically lower CR rates were associated with the largest tophi in both pegloticase groups (responders and nonresponders). Among responders at the week 13 visit, TT-CR rates were 76%, 54% and 8%, respectively, with biweekly pegloticase and 90%, 86% and 17%, respectively, with monthly pegloticase for tophi with a baseline area smaller than 250 mm^2^, 250 to 750 mm^2^ and tophi larger than 750 mm^2^. Corresponding values were 84%, 91% and 64% in the biweekly pegloticase group and 91%, 100% and 33% in the monthly pegloticase group for small, medium and large tophi at the patient’s final visit. A similar trend was observed in patients who received open-label pegloticase either biweekly or monthly after receiving placebo during the RCTs.

## Discussion

A total of 73% of patients enrolled in the RCTs of pegloticase had one or more tophi at baseline, a proportion that is consistent with published study populations defined by refractory gout [1,2]. Biweekly pegloticase—the regimen approved in the United States and European Union—resulted in complete resolution of at least one target tophus without development of any new tophi or progression of existing tophi in 22% of evaluable patients at three months (the first protocol assessment) and in 45% of patients at six months. The benefit of pegloticase on tophus burden increased with continued therapy during the OLE study, particularly among patients who responded to treatment with sustained PUA reductions during the RCTs. By the final OLE visit, 59% of all patients who received pegloticase during both the RCT and OLE study achieved the best overall tophus response of CR. Among the patients who received pegloticase in both the RCT and the OLE study and showed a sustained urate-lowering response (as defined by the primary endpoint of the RCT), the overall tophus CR rate exceeded 80%.

The phase III and OLE studies of pegloticase employed a standardized method (CAPER) for assessing tophus size. This methodology, based upon the Response Evaluation Criteria in Solid Tumors [18], was developed to provide categorical scoring of tophus response captured by digital images of subcutaneous tophi. Multiple methods of measuring tophi have previously been described. These methods include direct physical tophus measurements using tape measures [19] or calipers [11] and imaging modalities such as ultrasonography [20], magnetic resonance imaging [21-24], computed tomography (CT) [21,25,26] or dual-energy CT [27,28]. A systematic review [29] of tophus assessment methods aimed at determining their utility based on the Outcome Measures in Rheumatology (OMERACT) filter (feasibility, truth and discrimination) concluded that physical measurement techniques are most feasible and generally fulfill the OMERACT filters. The CAPER method is distinguished by the use of standardized digital photography, computer-assisted measurements, analysis by an independent reviewer and a blinded, central reader approach. Tophus responses in the pegloticase trials paralleled treatment regimens, SUA levels and treatment duration, thus demonstrating that the CAPER method showed sensitivity to change and between-group sensitivity [29]. Furthermore, CAPER provides several of the advantages of advanced imaging modalities (raw data storage and management, along with standardization using central readers) and is cost-effective, easy to implement and patient-friendly. CAPER may be a valuable tool for assessing tophus resolution in future clinical trials.

The rapidity of tophus resolution or reduction achieved with pegloticase stands in contrast to data derived from published trials of currently available urate-lowering agents, although it should be noted that comparisons between clinical trials with different populations and measurement methods have well-documented limitations. For example, in the pivotal phase III study evaluating febuxostat versus allopurinol, median reduction in tophus **area** (assessed by clinical examination) after 12 months of therapy was 83% with 80 mg of febuxostat, 66% with 120 mg of febuxostat and 50% with allopurinol [30]. No significant change was seen in the number of tophi over time in any of the treatment groups. Patients enrolled in the febuxostat study were not defined by chronic gout refractory to conventional therapy; rather, their diagnosis of gout was based on the identification of urate crystals in joint fluid, a tophus shown to contain urate crystals and/or the presence of at least six clinical, laboratory and X-ray characteristics described according to the 1977 preliminary criteria for gout published by the American Rheumatism Association [31].

To date, only pegloticase has demonstrated a significant impact on tophus burden in controlled trials. Prior to the approval of pegloticase, rasburicase, a non-PEGylated recombinant uricase not indicated for the treatment of gout, demonstrated tophus reduction and/or resolution within a similar time frame as pegloticase. In a small, retrospective, exploratory study, tophus resolution and/or reduction was reported in two of four patients with refractory tophaceous gout after six monthly infusions of rasburicase [32].

The rapid tophus resolution seen with pegloticase treatment may be explained by the profound and sustained lowering of urate levels. *In vitro* experiments have shown that the rate at which urate crystals dissolve is proportional to the degree of urate lowering in the surrounding fluid [33]. Investigators in a small serial ultrasound study recently reported that evidence of subacute crystal dissolution was seen only in patients with sustained SUA levels of 6 mg/dl or lower [34]. In a seminal study, Perez-Ruiz and colleagues observed an inverse relationship between mean SUA and the rate of decrease in tophus size in gout patients who received ULT [11]. Our data add to the implications of these findings by providing clinical evidence that tophus resolution can be achieved in select patients within several months if UA is reduced to levels well below what has been achieved to date with oral agents. This was apparent in the majority of patients showing sustained UA lowering with biweekly pegloticase treatment and also in the 26% of nonresponders, all of whom experienced initial but temporary reductions in UA. Tophus CR in such transient responders to pegloticase treatment suggests that UA reduction, even if short-lived, can be associated with resolution of some tophi.

Limitations of the RCTs and the OLE study have been described in their respective publications [16,35]. The CAPER methodology, used as a tool for evaluating tophi first reported in detail herein, has some limitations. First, although CAPER incorporates standardization and rigor by using bidirectional measurements and independent central readers, assessment was based on a two-dimensional image of a three-dimensional lesion. For example, the central reader was not able to assess the firmness of tophi or palpate borders. We believe that, because CR was the essential therapeutic goal, evaluation of subtle differences in borders was not needed for our analysis. Second, tophi identified by the central reader at baseline were not confirmed by clinical examinations. For two patients in the placebo arm, tophi were categorized as CR; it is likely that these lesions were not tophi, but rather areas of cystic nongouty lesions or joint inflammation that might have resolved after the baseline measurement. Third, photographic analyses were undertaken for the hands and feet and up to two other locations. Some patients had additional visible tophi at other sites at baseline, and resolution of these “nontarget” tophi was not captured. Thus, we may have underestimated the number of patients with complete resolution of baseline tophi.

## Conclusions

Pegloticase, at the approved dosing regimen of 8 mg every two weeks, produced sustained normalization of PUA levels to less than 6 mg/dl in 42% of patients. This improvement was associated with complete resolution of at least one tophus in 22% of these patients after 13 weeks of blinded therapy and in 45% after 25 weeks of therapy. Continued treatment with pegloticase resulted in progressively greater numbers of patients with a best overall tophus response of CR. This proportion reached 59% of patients by their final visit in the OLE study from among all patients who received pegloticase during both the RCT and OLE study. Importantly, tophus CR was achieved by more than 80% of patients who maintained PUA levels lower than 6 mg/dl for the duration of treatment. The results reported herein support pegloticase as an important disease-modifying therapy [16] for patients who have a significant and refractory tophus burden.

## Abbreviations

BMI: Body mass index; CAPER: Computer-Assisted Photographic Evaluation in Rheumatology; CR: Complete response; mITT: Modified intent-to-treat; MR: Marked response; OLE: Open-label extension; OMERACT: Outcome Measures in Rheumatology; PD: Progressive disease; PR: Partial response; PUA: Plasma uric acid; q2 weeks: every 2 weeks; q4 weeks: every 4 weeks; RCT: Randomized controlled trial; SD: Stable disease; SUA: Serum uric acid; TT-CR: Target tophus complete response; ULT: Urate-lowering therapy.

## Competing interests

HSBB, MAB, SRGU, ELT, JVM, JSS and RAY were investigators in the pegloticase trials. HSBB has indicated that he has received grant/research support from Ardea Biosciences, Metabolex Inc, Novartis Pharmaceuticals, Nuon Therapeutics, Regeneron Pharmaceuticals, Savient Pharmaceuticals, Inc and Takeda Pharmaceuticals. He is also a consultant for Takeda and Savient Pharmaceuticals and is on the speaker’s bureau for Savient Pharmaceuticals, Inc, and Takeda. MAB has indicated that he consults for and has received grant and research support from Savient Pharmaceuticals, Inc, and Takeda Pharmaceuticals and that he is a consultant for Ardea Biosciences, BioCryst Pharmaceuticals, Chugai Pharmaceuticals, Metabolex Inc, Mutual/URL Pharmaceuticals and Regeneron Pharmaceuticals. CDR is an employee of Savient Pharmaceuticals, Inc. FDO is a former employee of Savient Pharmaceuticals, Inc. JSS has indicated that he has received consulting fees from Savient Pharmaceuticals, Inc. He is a consultant for Ardea Biosciences, Regeneron Pharmaceuticals, Metabolex Inc and Pharmos. He receives research support from Ardea Biosciences, Metabolex Inc and Pharmos. RAY has indicated that he has received grant support from Takeda Pharmaceuticals.

## Authors’ contributions

HSBB, MAB, SRGU, ELT, JVM, JSS, and RAY were investigators in the pegloticase clinical trials, provided clinical expertise in the design and implementation of the studies and made substantial contributions to the data collection, analysis and interpretation. FDO and CDR contributed to the design and management of the clinical trials. All authors were involved in drafting the manuscript and critical review and revision at multiple draft stages. All authors read and approved the final manuscript.
